# Stabilization Methods for a Multiagent System with Complex Behaviours

**DOI:** 10.1155/2015/236285

**Published:** 2015-05-13

**Authors:** Florin Leon

**Affiliations:** Department of Computer Science and Engineering, “Gheorghe Asachi” Technical University of Iaşi, D. Mangeron 27 Street, 700050 Iaşi, Romania

## Abstract

The main focus of the paper is the stability analysis of a class of multiagent systems based on an interaction protocol which can generate different types of overall behaviours, from asymptotically stable to chaotic. We present several interpretations of stability and suggest two methods to assess the stability of the system, based on the internal models of the agents and on the external, observed behaviour. Since it is very difficult to predict a priori whether a system will be stable or unstable, we propose three heuristic methods that can be used to stabilize such a system during its execution, with minimal changes to its state.

## 1. Introduction

Multiagent systems have enjoyed increasing popularity by both researchers and practitioners, due to the continuation of trends in computing such as ubiquity, interconnection, intelligence, delegation, and human-orientation [1]. Agent-based systems can be used as a tool to understand social and economic systems, since the aggregation of simple rules of behaviour for individual agents often leads to an extremely rich range of emergent behaviours, which can help to understand complex phenomena in human societies [2–12].

The actions of individual agents are often based on a certain process of decision making. Therefore, stability, intuitively understood as the property of a system to exhibit bounded behaviour [13], is one of the most desired features in multiagent systems, because of the importance of predicting their response to various conditions in the environment.

The initial motivation of developing the interaction protocol which will be analysed in the present paper was to design a set of simple interaction rules which in turn can generate, through a cascade effect, different types of overall behaviours, that is, stable, periodical, quasi-periodical, chaotic, and nondeterministically unstable. They can be considered metaphors for the different kinds of everyday social or economic interactions, whose effects are sometimes entirely predictable and can lead to an equilibrium while, in some other times, fluctuations can widely affect the system state, and even if the system appears to be stable for long periods of time, sudden changes can occur unpredictably because of subtle changes in the internal state of the system.

We organize our paper as follows. In Section 2, we review some of the related work in the areas of dynamical and evolutionary behaviours, stabilization, and phase transitions in multiagent systems. In Section 3, we describe the interaction protocol employed by the multiagent system under study. In Section 4, we present different interpretations of stability and suggest two methods to assess the stability of the system, based on the internal model and the external, observed behaviour. In Section 5, we give some empirical evidence about the difficulty of predicting a priori whether the system will be stable or unstable and present experiments that can help to identify the nature of phase transitions.  Section 6 describes three heuristic methods for the stabilization of the system with minimal changes, along with case studies and discussion.  Section 7 contains the conclusions of the paper and addresses some future lines of investigation.

## 2. Related Work

An important aspect of dynamical systems is the issue of stability. In the multiagent systems literature, the study of stability is mainly applied for three broad types of problems. The first is the question of stability in evolutionary systems. The second is formation control, including the stability of simulated swarms of particles or groups of mobile robots. The third is the general issue of convergence of agent interactions, consensus in networks of agents modelled using graphs, and so forth.

Nonlinear effects are characteristic of evolutionary game theory [14], which aims to enhance the concepts of classical game theory [15] with evolutionary issues, such as the possibility to adapt and learn. In general, the fitness of a certain phenotype is, in some way, proportional to its diffusion in the population. The strategies of classical game theory are replaced by genetic or cultural traits, which are inherited, possibly with mutations. The payoff of a game is interpreted as the fitness of the agents involved [16].

Many such models have been proposed, based on the different ways in which agents change their behaviours over time. Among them we can mention replicator dynamics [17, 18], its replicator-mutator generalization [19], and the quasi-species model [20], which have been used to model social and multiagent network dynamics [21–23].

The emergence of cooperation within groups of selfish individuals, where cooperators compete with defectors, is an interesting research direction because it may seem to contradict natural selection. Studying the evolutionary stability in games involving cooperative and noncooperative behaviours such as the repeated prisoners' dilemma, a set of conditions were found which enable the emergence of cooperation and its evolutionary stability [24]. Recent results reveal that the evolution of strategies alone may be insufficient to fully exploit the benefits of cooperative behaviour and that coevolutionary rules can lead to a better understanding of the occurrence of cooperation [25].

Cyclic interactions, which occur in simple games such as rock-paper-scissors, emerge spontaneously in evolutionary games entailing volunteering, reward, and punishment and are common when the competing strategies are three or more regardless of the particularities of the game. Cyclic dominance is also a key to understanding predator-prey interactions or mating and growth strategies of biological organisms. A review of these issues [26] also presents an analysis of stability of spatial patterns that typically emerge in cyclic interactions.

Another study of the group interactions on structured populations including lattices, complex networks, and coevolutionary models highlights the synergic contribution of statistical physics, network science, and evolutionary game theory to the analysis of their dynamics [27]. In general, it is considered that group interactions cannot be reduced to the corresponding sum of pairwise interactions.

The evolution of public cooperation on complex networks is particularly important and has been studied, for example, in the context of public goods games [28] or the emergent behaviour of agent social networks [29].

In the context of diffusion, which allows players to move within the population, the analysis of the spatiotemporal patterns reveals the presence of chaos, which fits the complexity of solutions one is likely to encounter when studying group interactions on structured populations [30].

The applicability of the concept of evolutionary games can be found in social and natural sciences, with examples such as an RNA virus [31], ATP-producing pathways [32], and traffic congestion [33].

By using ideas from evolutionary computing, a multiagent system can be seen as a discrete Markov chain and its evolution as a Markov process, possibly with unknown transition probabilities [13]. In a model using this approach, in which the number of agents varies according to the fitness of the individuals, a definition for the degree of instability is proposed based on the entropy of the limit probabilities [34].

In the second category, some authors analysed the stability of small groups of fully interconnected particles [35–37]. A centralized algorithm for a system of particles that leads to irregular collapse and a distributed algorithm that leads to irregular fragmentation were proposed [38]. A universal definition of flocking for particle systems with similarities to Lyapunov stability was also suggested [39].

The stability of formation control was analysed in terms of the limitations of the number of communication channels, showing that stability is maintained by appropriately constructing the switching sequence of network structure [40]. Stable flocking motion can be obtained using a coordination scheme that generates smooth control laws for particles or agents, based on attractive and repulsive forces, and stability is investigated with double integrator dynamics [41]. The coordinated control of mobile robots where the agents are periodically interconnected leads to the formulation of a theoretical framework in which the stability of many distributed systems can be considered [42]. For the problem of formation control of a group of homogenous agents, necessary and sufficient conditions for stability in case of arbitrary time-invariant communication topologies were proposed, which reduced the analysis of multiagent systems with any number of agents to the analysis of a single agent [43].

Conditions for swarm stability of nonlinear high-order multiagent systems were also described based on the idea of space transformation, showing that swarm stability can be ensured by sufficient connectivity of graph topology and dissipative property regulated by relative Lyapunov function, with two independent variables, for time-varying or heterogeneous models [44].

Distributed control strategies for motion coordination were proposed and demonstrated to work for the rendezvous problem of planar roving agents using contraction theory. It was found that even if noise were present in the network, rendezvous would still be achieved with some final bounded error because of the contractivity of all the agents guaranteed by the protocol [45]. The rendezvous problem is related to the classical consensus problem in networks and therefore can be solved using a similar approach.

For systems that belong to the third category, stability was analysed for the problem of iterative contracting of tasks. The interactions were resource reallocations through a memoryless bidding protocol where every agent calculated its next bidding price using a discount drawn from a Gaussian distribution. The results showed that the network of agents reached an equilibrium distribution, even in the presence of noise [46].

Another model of a network of agents interacting via time-dependent communication links was proposed which can be applied in domains such as synchronization, swarming, and distributed decision making. Each agent updates its current state based on the current information received from its neighbours. It was shown that, in case of bidirectional communication, convergence of the individual agents' states to a common value is guaranteed if, during each (possible infinite) time interval, each agent sends information to every other agent, either through direct communication or indirectly via intermediate agents. In case of unidirectional communication, convergence is proven if a uniform bound is imposed on the time it takes for the information to spread over the network [47].

In a scenario in which leaders are required to recruit teams of followers, satisfying some team size constraints, and where agents have only local information of the network topology, an algorithm was proposed and shown to converge to an approximate stable solution in polynomial time. For general graphs, it was found that there can be an exponential time gap between convergence to an approximate solution and convergence to a stable solution [48].

The problem of coordination in multiagent systems becomes more difficult when agents have asynchronous, uncertain clocks. For this situation, necessary and sufficient conditions for stability were suggested based on linear matrix inequalities. The analysis was applied to networked control with random sampling times, as well as an asynchronous consensus protocol. The stability of linear multiagent systems with noisy clocks was also studied [49].

In case of a supervisory control scheme that achieves either asymptotic stability or consensus for a group of homogenous agents described by a positive state-space model, necessary and sufficient conditions for the asymptotic stability, or the consensus of all agents, were derived under the positivity constraint [50].

Some approaches are hybrid, for example, trying to obtain stabilizing control laws for problems such as state agreement with quantized communication and distance-based formation control. Stabilizing control laws are provided when the communication graph is a tree [51].

For heterogeneous, interconnected multiagent systems where the interaction topology is an arbitrary directed graph, a general method was proposed to derive the transfer functions between any pair of agents with different dynamics. A class of multiagent systems is presented for which a separation principle is possible, in order to relate formation stability to interaction topology [52].

Another important issue when analysing the behaviour of a dynamical system is the presence of phase transitions. A phase transition in a system refers to the sudden change of a system property when some parameter of the system crosses a certain threshold value. This kind of changes has been observed in many fields, such as physics, for example, Ising magnetization model [53, 54], graph theory, for example, random graphs [55], cellular automata [56, 57], or biology, for example, the evolution of the numbers of two species [58]. Phase transitions have been observed in multiagent systems displaying simple communication behaviour, that is, when agents update their states based on the information about the states of their neighbours received under the presence of noise [59]. It was shown that, at a noise level higher than some threshold, the system generates symmetric behaviour or disagreement, whereas, at a noise level lower than the threshold, the system exhibits spontaneous symmetry breaking or consensus.

Our proposed multiagent system described in Section 3 also belongs to the third category but exhibits certain differences from other existing models.

## 3. Description of the Multiagent Interaction Protocol

The main goal in designing the structure and the interactions of the multiagent system was to find a simple setting that can generate complex behaviours. A delicate balance was needed in this respect. On the one hand, if the system is too simple, its behaviour will be completely deterministic. On the other hand, if the system is overly complex, it would be very difficult to assess the contribution of the individual internal components to its observed evolution. The multiagent system presented as follows is the result of many attempts of finding this balance. The wide range of observed behaviours from stable and periodic to chaotic and nondeterministically unstable was described in two previous papers [60, 61].

The proposed system is comprised of *n* agents; let *A* be the set of agents. Each agent has *m* needs and *m* resources, whose values lie in their predefined domains *D*_*n*_, *D*_*r*_ ⊂ *ℝ* ^+^. This is a simplified conceptualization of any social or economic model, where the interactions of the individuals are based on some resource exchanges, of any nature, and where individuals have different valuations of the types of resources involved.

It is assumed that the needs of an agent are fixed (although it is possible to consider an adaptive mechanism [62, 63] as well), that its resources are variable, and that they change following the continuous interactions with other agents.

Also, the agents are situated in their execution environment: each agent *a* has a position *π*_*a*_ and can interact only with the other agents in its neighbourhood Λ_*a*_. For simplicity, the environment is considered to be a bidimensional square lattice, but this imposes no limitation on the general interaction model; it can be applied without changes to any environment topology.

### 3.1. Social Model

Throughout the execution of the system, each agent, in turn, chooses another agent in its local neighbourhood to interact with. Each agent *a* stores the number of previous interactions with any other agent *b*, *i*_*a*_(*b*), and the cumulative outcome of these interactions, *o*_*a*_(*b*), which is based on the profits resulting from resource exchanges, as described in the following section.

When an agent *a* must choose another agent to interact with, it chooses the agent in its neighbourhood with the highest estimated outcome: *b* ^∗^ = arg max⁡_*b*∈Λ_*a*__ *o*_*a*_(*b*).

The parallelism of agent execution is usually simulated by running the agents sequentially and in random order. Since one 3 of the goals of the system is to be deterministic, we define the execution order from the start. Thus, at any time, it can be known which agent will be active and with which other agent the active agent will choose to interact. In our case the random order is not necessary to generate complex behaviours. Even if the agents are always executed in lexicographic order (first A1, then A2, then A3, etc.), sudden changes in utilities still occur, although the overall aspect of the system evolution is much smoother.

### 3.2. Bilateral Interaction Protocol

In any interaction, each agent tries to satisfy the needs of the other agent as well as possible, that is, in decreasing order of its needs. The interaction actually represents the transfer of a resource quantum *γ* from an agent to the other. Ideally, each agent would satisfy the greatest need of the other.

For example, let us consider 3 needs (*N*) and 3 resources (*R*) for 2 agents *a* and *b*: *N*_*a*_ = {1,2, 3}, *N*_*b*_ = {2,3, 1}, *R*_*a*_ = {5,7, 4}, *R*_*b*_ = {6,6, 5}, and *γ* = 1. Since need 2 is the maximum of agent *b*, agent *a* will give *b* 1 unit of resource 2. Conversely, *b* will give *a* 1 unit of resource 3.

In order to add a layer of nonlinearity, we consider that an exchange is possible only if the amount of a resource exceeds a threshold level *θ* and if the giving agent *a* has a greater amount of the corresponding selected resource *r*_sel_ than the receiving agent *b*: *R*_*a*_(*r*_sel_) > *R*_*b*_(*r*_sel_) and *R*_*a*_(*r*_sel_) > *θ*.

In the previous situation, if we impose a threshold level *θ* = 5, agent *a* will still give *b* 1 unit of resource 2, but *b* will only satisfy need 1 for agent *a*.

Based on these exchanges, the resources are updated and the profit *p*_*a*_ is computed for an agent *a* as follows:

(1) $$\begin{array}{c} p_{a}=\gamma \cdot N_{a}\left(\;r_{\text{sel}}\;\right)\cdot \frac{R_{b}\left(r_{\text{sel}}\right)}{R_{a}\left(r_{\text{sel}}\right)}. \end{array}$$

A bilateral interaction can bring an agent a profit greater than or equal to 0. However, its utility should be able to both increase and decrease. For this purpose, we compute a statistical average of the profit, *p*_avg_, increase the utility of an agent if the actual profit is above *p*_avg_, and decrease the utility if the profit is below *p*_avg_.

Thus, the equation for updating the utility level of an agent *a* is

(2) $$\begin{array}{c} u_{a}⟵\frac{u_{a}\cdot i_{a}^{\text{adj}}+\eta \cdot \left(p_{a}-p_{\text{avg}}\right)}{i_{a}^{\text{adj}}+1}, \end{array}$$

where the adjusted number of interactions is *i*_*a*_^adj^ = min⁡(∑_*b*∈*A*_ *i*_*a*_(*b*), *i*_mem_), *i*_mem_ is the maximum number of overall interactions that the agent can “remember” (i.e., take into account), and *η* is the rate of utility change. At the beginning, the utility of the agent can fluctuate more, as the agent explores the interactions with its neighbours. Afterwards, the change in utility decreases but never becomes too small.

Similarly, the social outcome of an agent *a* concerning agent *b* is updated as follows:

(3) $$\begin{array}{c} o_{a}\left(\;b\;\right)⟵\frac{o_{a}\left(b\right)\cdot i_{a}\left(b\right)+\eta \cdot \left(p_{a}-p_{\text{avg}}\right)}{i_{a}\left(b\right)+1}. \end{array}$$

In this case, the social model concerns only 1 agent and thus the use of the actual number of interactions can help the convergence of the estimation an agent has about another.

Regarding the computation of the average profit, a global parameter of the system, we used a statistical approach where we took into account 1000 continuous interactions between two randomly initialized agents, which exchange resources for 100,000 time steps. The average profit depends on the number of resources, their domain, and the interaction threshold.

Since the social outcome depends on the average profit, the latter must be a fixed point of the corresponding system. If the initial value *p*_avg_^0^ < *p*_avg_, the result of the simulation will provide a value *p*_avg_^sim^ > *p*_avg_^0^ and vice versa. Therefore the correct value can be iteratively approximated by increasing or decreasing *p*_avg_^0^ until *p*_avg_^0^ ≈ *p*_avg_^sim^ ≈ *p*_avg_.

In the following sections, we will present some heuristic methods to stabilize the utility functions of the agents, such that they reach an equilibrium, in a minimally invasive way, that is, with the least amount of change to the system. We will explore the effect of small endogenous (internal) perturbations, in terms of alternative decisions made by the agents, to the stability of the multiagent system.

## 4. Interpretations of Stability

### 4.1. Statistical and Game Theoretical Interpretations

From a statistical point of view, the evolution of the multiagent system can be viewed as a Markov process with an initial distribution and transition matrix. The state of the system at time step *n* is represented by a random variable *X*_*n*_, which is a vector that contains the probabilities of certain parameters that define the actual state of the system (e.g., agent locations, utilities, and characteristics). In this case, the system can be considered to be stable if the distribution of states converges to an equilibrium distribution; that is, *P*(*X*_*n*_ = *j*) → *π*_*j*_, when *n* → *∞*. In other words, the system is stable if the probability distribution of system states becomes independent of the time step *n*, for large values of *n* [13].

However, this interpretation cannot be easily applied for the proposed multiagent interaction protocol. First, the visible output of the system is continuous, but it could be discretized into a convenient number of states. Secondly, all the interactions in the system are deterministic; while it is possible to make a statistical interpretation of a larger number of behaviours, the probabilistic framework would generate only approximations of the real evolution of a particular configuration. Thirdly, the most important aspect is that the accumulation of the nonlinear effects makes it very difficult to estimate the transitions between different states, because these transitions do not depend on the decisions of one agent at a time but on the aggregation of the decisions of all agents involved.

Stability is a relatively well-understood concept in physics, where it is regarded as a property of a stable equilibrium, that is, a state where the system returns on its own after a small perturbation. For multiagent systems, stability can also be defined with respect to perturbations of some sort, such as noise, variations in parameter values, or addition or removal of agents. If a small initial disturbance later becomes significant, the system is considered unstable. A multiagent system can be considered in equilibrium when the statistical properties of its performance indicators remain stationary when the external conditions that can impact the system vary [46].

In our case, it has been demonstrated that small perturbations can sometimes change the final state entirely, while in other cases the perturbations fade away [60, 61]. In the present paper, we use small perturbations to correct instability, such that the behaviour of the system should become easier to predict.

Multiagent systems can also be seen as a set of agents engaged in a multiplayer game. In game theory, stability is also a property of equilibrium, and the problem of finding an equilibrium is equivalent to the choice of an optimal strategy. Stability can then be used to describe the characteristics of the set of strategies in equilibrium, where the strategy of each player is the best response to the strategy of the others and where no player has any incentive to deviate, for example, Nash equilibrium [64].

Another related definition of stability considers the set of all actions performed by all agents in the system. An equilibrium point is one where no agent needs to further operate within the system and accepts the currently reached state. A set of actions, by the different agents, is stable if, assuming that an oracle could inform each agent about all the actions in the set performed by the other agents (and all the external events in the environment), each agent would do exactly what is in the set; that is, its observable behaviour would be exactly what the set predicts [65].

Considering again our multiagent system, the main difference in a game theoretic approach is that we take into account the observed behaviour and not the decisions that agents make. We need methods to find an equilibrium in an implicit not explicit way by considering the dynamic succession of interactions between agents.

Therefore, in the following sections, we try to identify some definitions of stability which are more appropriate for our multiagent system.

### 4.2. External Behaviour Interpretation

**Figure 1:**
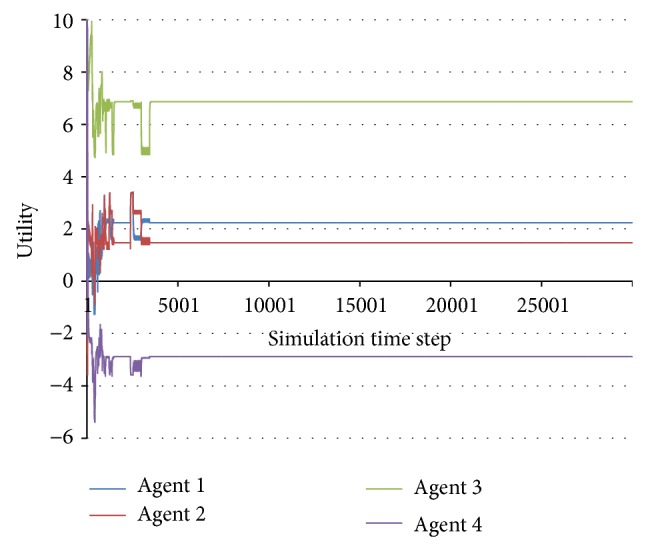
Stable behaviour.

From the practical point of view, stabilization can be defined in a straightforward way as the lack of further change to the agent utilities, starting from a certain time step of the simulation (Figure 1).

As described in the previous works [60, 61], the multiagent system under analysis can exhibit many types of behaviours, such as being stable, periodical, chaotic, and nondeterministically unstable. However, in the rest of this paper, we will only distinguish between stable and unstable behaviours. Small periodic oscillations of the utility function will also be considered to represent stable behaviour.

**Figure 2:**
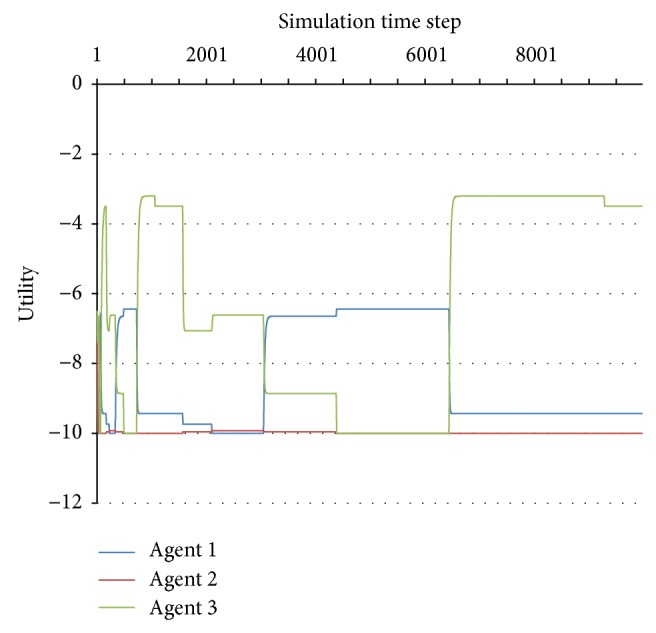
Unstable behaviour.

When the system exhibits unstable behaviour (either deterministically chaotic or nondeterministically unstable), the utilities of the agents continue to exhibit changes throughout the simulation, with no clear horizon of becoming constant. An example of unstable behaviour is presented in Figure 2.

The stability of the system can be considered in a statistical way. A simple analysis has been made [61] showing that the probability of instability increases as the number of agents in the system increases. This is not surprising, because instability clearly depends on the complexity of agent interactions. However, when we consider the multiagent system at a lower level, we see that instability can occur even with as little as 3 agents; therefore, in the following, we aim at understanding the internal conditions that cause the overall instability.

From an external point of view, it is not difficult to distinguish between stability and instability. The procedure is the following. We first set an arbitrary number of simulation steps to analyse the behaviour. In a previous paper [61], we chose 10 million steps to observe whether an unstable behaviour eventually stabilizes, and it was found that it continues to exhibit sudden changes even after long periods of stability.

Then, since at the beginning of the simulation there is usually a transitory period with many changes, in considering the question of stability one should ignore this transitory unstable period.

In between these two time limits, one can observe the evolution of the utility functions. If the absolute difference between the minimum and maximum values of the utility function of the agents exceeds a certain threshold (e.g., 0.1 or 1), then the behaviour is considered to be unstable.

Another issue is the presence of periodic oscillations. Small oscillations are very often present in case of systems with a larger number of agents (e.g., above 6). In order to eliminate them and consider this type of behaviour stable, we can use a moving average window and smoothen the utility functions.

**Figure 3:**
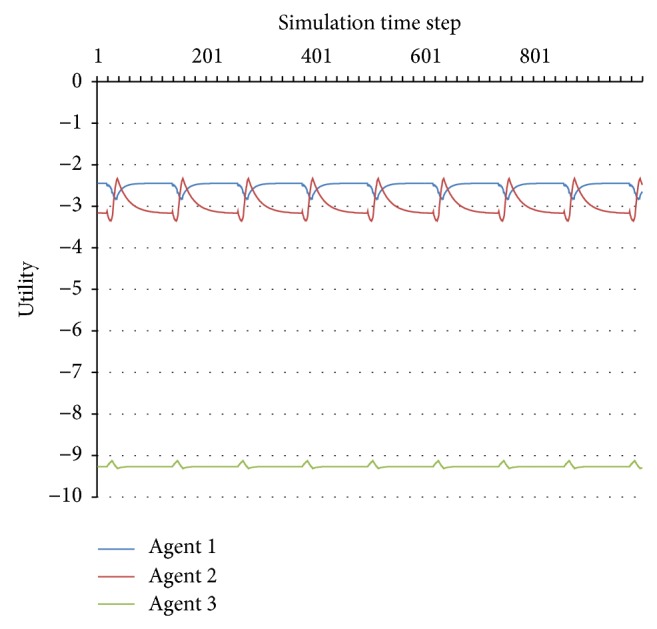
Unstable periodic behaviour.

**Figure 4:**
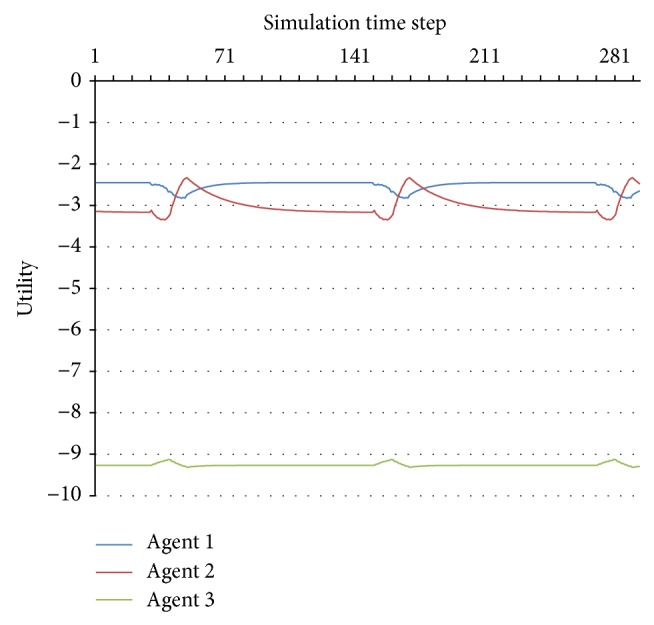
Close-up of the unstable periodic behaviour.

This method cannot be applied for complex periodic behaviours, such as the one presented in Figures 3 and 4. However, since this kind of behaviour is predictable, we will also consider it to be stable.

Thus, our focus for stabilization is on the aperiodic behaviours. Although with different probabilities, this type of unpredictable evolution can appear to any number of agents greater than 2. The reason why 2 agents cannot exhibit unstable behaviour is explained in the next section.

### 4.3. Internal Model Interpretation

While obviously useful, the external perspective of the system fails to address two fundamental questions of stability: why sudden changes can appear after long periods of stability and even if the system appears to be stable during the time period considered for analysis, what guarantees we have that it will not exhibit large changes again, sometime after the upper time limit of the simulation.

The answers to these questions can be found only by opening the “black box” of the internal social models of the agents.

Stability appears when the interactions of the agents follow a certain structure; for example, an agent *a* keeps on interacting with an agent *b*, because *b* brings *a* maximum profit. This situation is reflected in the social model of agent *a*, and thus *a* chooses *b* because *b* has the largest social outcome in agent *a*'s internal model (according to (3)). If all the agents keep interacting with the same peers, the profits they receive (1) are the same in every time step and their utility functions quickly converge (2).

The utility function of an agent changes when the profit it receives in one step is different from the profit it has received in the previous step. This is caused by the nonlinearity in the way in which resources are given. A resource unit is given only when the amount that an agent has exceeds a certain threshold (e.g., 5). But when the agents keep interacting with the same peers in a stable way, the amount of resources an agent has at the beginning of a time step remains constant: a resource unit is passed between the agents in a neighbourhood until it gets back to the first agent.

However, the “opinion” an agent has about its peers is highly dynamic. The social outcome of the best agent to interact with can decrease. For a while that agent remains the best option. But at some point, the value of its social model decreases below the one of another agent. This causes a shift in the interaction pattern of an agent. The “second best” now becomes the “best.” The interaction with this new agent can be better or worse than the interaction with the “old best.” Some of its resources which the agent who initiates the interaction desires may be below the threshold and cannot be provided. This happens especially when the number of resources is small (e.g., below 3). Otherwise, agents can usually find a resource to give. But it can nevertheless bring about a lower profit to the receiver.

When the interaction is not profitable enough, the value of the social model of the “best option” again decreases, and soon another agent will be selected for interaction (either the previous best or a completely different one).

In this way, we can see that the changes in the social ranking of agents are the internal causes for instability.

**Figure 5:**
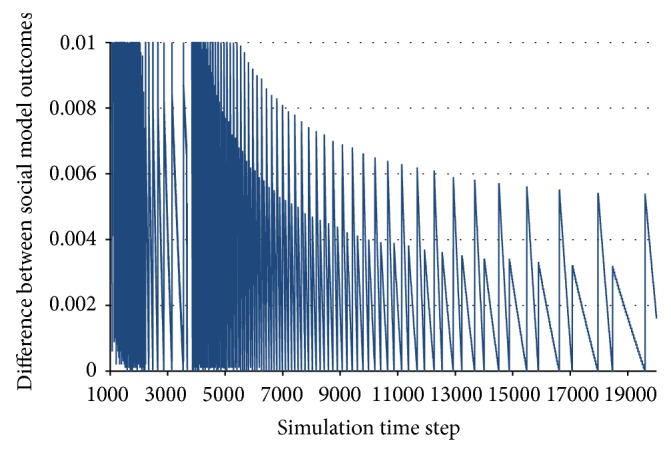
Internal changes in the social model leading to unstable behaviour.

In order to represent the internal changes more efficiently, especially when the number of agents is large, we can take into account the difference between the social outcome of the best peer and the social outcome of the second best. When the best becomes less attractive, the difference will decrease and a change will occur after it reaches 0. Let us consider the example with an unstable behaviour. We can see that all agents follow the same pattern of temporary stability and changes. Among them, Agent 4 has the lowest amplitude of these fluctuations. However, its internal model of the difference between its best and second best peer is displayed in Figure 5. We can see that the changes in all utility functions occur precisely when the difference in the social models of an agent reaches 0 and thus the agent's interaction patterns change. Although Agent 4 is not really affected by this phenomenon, the changes greatly impact the other agents. Basically, when an agent changes its preferred interaction partner, an external change occurs in its utility and/or the utility of other agents. If the ranks of the internal models keep changing, this will be reflected in the observed instability of the system.

**Figure 6:**
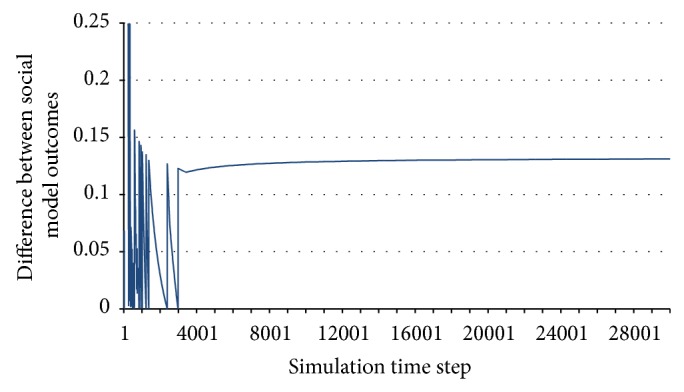
Internal changes in the social model leading to stable behaviour.

On the other hand, let us now consider a situation with a stable behaviour. After some initial fluctuations in a transitory phase, an agent finds a peer with whom the interactions are rewarding. The difference in the social model outcomes between the best and the second best monotonously increases (although at some point it seems to converge), as displayed in Figure 6. The same thing happens to the other agents as well. In this case, we have a guarantee that the considered system is stable and will remain stable forever, since no changes can occur any longer.

**Figure 7:**
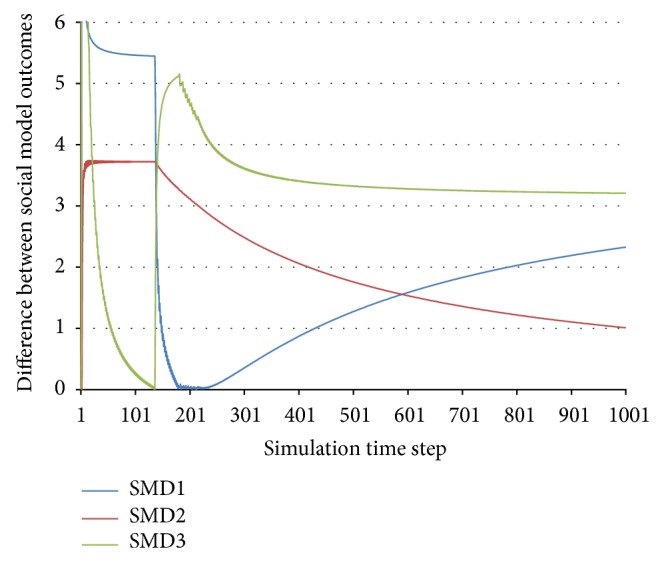
Interaction of changes in social model outcomes.

As stated above, the swaps in any agent's social model can affect the system, and since they can simultaneously occur in more agents, especially in large systems, their effects can be combined in ways which are difficult to predict analytically. For example, let us consider a system with 3 agents, whose differences in the social model outcomes are displayed in Figure 7. As before, these values represent the difference between the social model outcome of the best peer, which seems to bring about the greatest profit to the considered agent, and the social model outcome of the second best peer. The 3 functions in the figure represent the way in which the difference varies for each of the 3 agents considered in this case.

**Figure 8:**
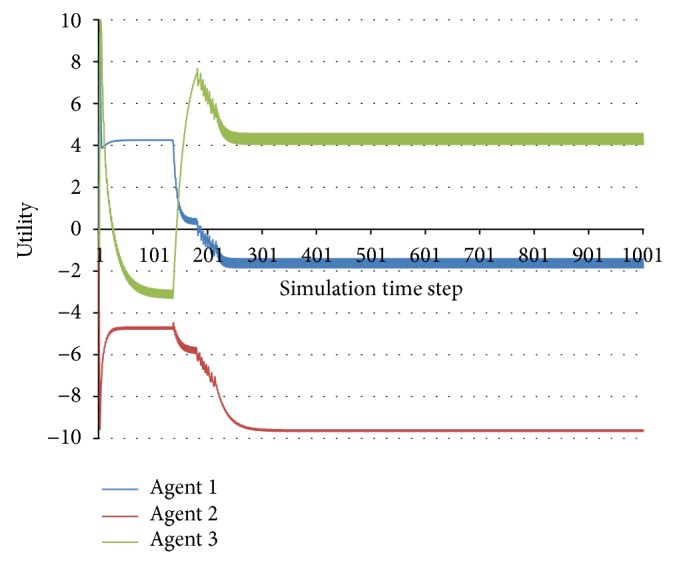
Observed behaviour when changes in the social model outcomes interact.

One can notice two swaps that interact: the first in time step 136 for Agent 3 and the second, with certain oscillations, in time steps 183–229 for Agent 1. The consequence can be observed in Figure 8, especially in the utility functions of Agent 1 and Agent 2: their utility functions have a particular evolution until time step 136, a different one between 136 and 183, another oscillatory one between 183 and 229, and another shape after time step 229.

The internal perspective can provide satisfactory answers to the questions we introduced at the beginning of the section. Since the difference between the best and second best can decrease very slowly, we can have long periods of stability. However, once a swap occurs, the utility functions change and this can lead to highly unstable behaviour. Also, the swap can occur after the time limit of the simulation. The system will be stable for the considered period of time but could prove to be actually unstable if the analysed time period were extended. Of course, it could stabilize soon after, as well. Also, this is why the systems with only 2 agents are always stable: because each agent has only one social model and swaps cannot occur during execution.

The only guarantee identified so far is when the differences in the social models are nondecreasing. However, it was empirically observed that this rarely happens, especially in systems with a large number of agents. Therefore, we could consider that most systems are inherently unstable. From the practical point of view, the external stability for a (possibly extended) period of time remains a good way to assess stability. The main goal of stabilization would be to make the system stable in the “strong,” internal sense; however since it may not be possible in general to do so, we can restrict the notion of stability to a “weaker” interpretation, dealing with the external observed behaviour.

### 4.4. Combining External and Internal Perspectives

In this section, we propose a method to check the stability of the multiagent system, taking into account both internal and external factors. The main idea is the following. We test whether the internal models of all the agents have a periodic evolution. If this is not the case, the system cannot be considered stable. Likewise, if the external evolution of the utility functions is not periodic, the system is not stable. If both the internal models of interactions and the external utility functions are periodic, we compute the period of the whole system as the least common multiple of all agents' periods. The overall period will be 1 if the system has converged into a stable state with constant utility functions. As stated before, by stability we also understand predictability, and thus a periodic system is considered under this assumption to be stable, although it is not constant. Finally, the differences in the social models outcomes are evaluated, and if the difference decreases for at least one agent, it is assumed that it will eventually reach 0 and cause a swap, and therefore the system is considered unstable. The method is presented in Pseudocode 1.

In Pseudocode 1, it is considered that an agent has a record of its recent history (e.g., the last 100 steps or more), in terms of the agents with which it has interacted, what resources have been exchanged, and the values of its utility function. PeriodicModel checks whether an agent has been involved in periodic interactions. PeriodicUtility checks whether the values of its utility function are periodic. In case of a periodic behaviour, modelPeriod and utilityPeriod contain the actual values. Since they can be different, the period of the overall system is assessed as the least common multiple of all periods of the individual agents, both internal and external. modelDifference represents the difference between the best social model outcome of an agent and the second best one. If this difference decreases, it will eventually become zero and will cause a swap and thus an unstable behaviour.

It is important to say that the test for stability should be applied only after some initial time steps, which constitute the transitory period. In the beginning, all systems show fluctuations and therefore this initial period is not relevant to assess stability. The length of the transitory period depends on the particular multiagent system but is usually longer when the system is comprised of more agents.

A less restrictive variant of the stability test is to consider only the external, observed behaviour. From the practical point of view, if the simulation has a finite number of steps, we could be interested in finding a way to predict the overall system performance only for the specified period of time. Thus, an external test for stability is presented in Pseudocode 2, which will be later used in two heuristic methods, when another criterion based on the hybrid test fails to be satisfied.

This procedure is actually a simplified version of Pseudocode 1. It no longer contains the internal perspective test which checks whether the internal models are periodic. Pseudocode 1 also takes into account the interaction history of the agents and if the model difference (between the best peer and the second best peer) decreases, the system is considered to be unstable. Pseudocode 2 only checks the external perspective, that is, whether the utilities of the agents are periodic. Constant utilities, the most commonly encountered case of stability, are also included in this category, with a period equal to 1.

## 5. Difficulty of Predicting Stability

### 5.1. Classification Methods

In order to identify the nature of the multiagent system behaviours, we attempted to use classification methods to determine whether a system will be stable or unstable, based on its initial conditions. For this analysis, we used* Weka* [66], a popular collection of machine learning algorithms.

There are many classification algorithms available at the moment. For our analysis, we used four representative ones, which usually exhibit very good performance.

*C4.5* [67] generates a decision tree by recursive partitioning of data. The algorithm considers all the possible attribute tests that can split the data within a node and chooses the test that gives the best information gain, that is, the partitioning that would result in the most homogeneous child nodes. It can handle both symbolic and numerical attributes. The algorithm supports tree pruning at the end of the training process, which cuts off some parts of the tree in order to avoid overfitting.

The random tree classifier builds a tree that considers *k* random features for each node and performs no pruning; therefore its error rate on the training set alone is rather small. A* random forest* [68] is composed of several random trees. To classify a new instance, the input vector is presented to each of the trees in the forest. Each tree provides a classification, and its decision is considered to be a “vote” for that class. The forest chooses the class with the most votes. The method tries to avoid overfitting by using bagging to build individual trees for slightly different data.

The *k-nearest neighbour* (*k*NN) algorithm reduces the learning effort by simply storing the instances and classifying new ones on the basis of their closeness to their “neighbours,” that is, instances in the training set with similar attribute values. The nearest neighbour (NN) algorithm classifies a new instance in the same class as the closest stored instance in the attribute space. A straightforward extension is the *k*NN, where *k* neighbours, instead of 1, are taken into account. This approach is especially useful when the data are affected by noise and the nearest neighbour of an instance may indicate an erroneous class. Another optimization is to use distance weighting, that is, to give more importance to the neighbours which are closer to the instance to be classified.

*Support vector machines* [69] (SVM) are one of the best classification methods presently available. They consider the training instances as points in a multidimensional space, which can be transformed in order for the classes to become separable with a large margin. The transformation is performed by means of a function known as the kernel, for example, linear, polynomial, and radial basis functions. They also include a mechanism to reach a balance between good performance on the training set and the risk of overfitting, by allowing training instances to be misclassified by using a “soft margin,” with the intention of providing a simpler model which could generalize better.

For the classification of stable or unstable systems, the simplest case is to consider 3 agents and only 1 resource. Consequently, each agent will have only 1 need. Using the test for stability introduced in Section 4.4, we generated a training dataset of 1000 instances corresponding to 560 stable and 440 unstable systems. The dataset was created by generating different systems with random initial conditions (i.e., the values for the resources and needs). In order to maximize the interactions, here and in all subsequent case studies, all the agents are considered to have the same position, although the general model allows them to be distributed in the environment and interact only with the peers in their neighbourhood.

The classification results, in terms of accuracy obtained for the training set and by using 10-fold cross-validation, are presented in Table 1.

We can notice that only the training set enjoys good performance. No algorithm proves good generalization capabilities. The decision tree cannot learn a good model even on the training set. Random Forest and *k*NN overfit the data. SVM has the best generalization performance, but it is still far from being satisfactory.

In another case study we considered 10 agents and 10 resources, and the training set contains 1000 instances with a certain degree of imbalance: 742 stable and 258 unstable systems.  Table 2 shows the accuracy of the classification algorithms.

Except for C4.5, all the other techniques approximate the training set very well but perform poorly on cross-validation. Since the results of *k*NN and SVM with RBF kernel are identical, another type of kernel was also tried, the polynomial kernel of the second degree, which actually has lower generalization capability, although the error on the training set is 0. The reason for these results is that the data have a very high dimensionality (200 dimensions). Therefore, the two classes of training instances are very easily separable. However, this does not guarantee good generalization. It can be seen that *k*NN and SVM with RBF kernel have an error rate of 74.2%, which is exactly the percentage of stable instances. They actually fail to identify the characteristics of the unstable instances at all and classify them as being stable. The other algorithms perform even worse, with additional errors for the stable instances as well.

These results are not surprising, because the agents within the system are tightly coupled and the effect of their local interactions propagates throughout the simulation. Small changes accumulate and can affect the overall behaviour later on, in ways which are difficult or even impossible to predict beforehand. Moreover, since some behaviours of the system are chaotic [61], long-term prediction in such systems is impossible by definition. Therefore, we can conjecture that the stability (or instability) of the proposed interaction protocol is an emergent property and cannot be predicted only by analysing the initial static state of the agents.

**Figure 9:**
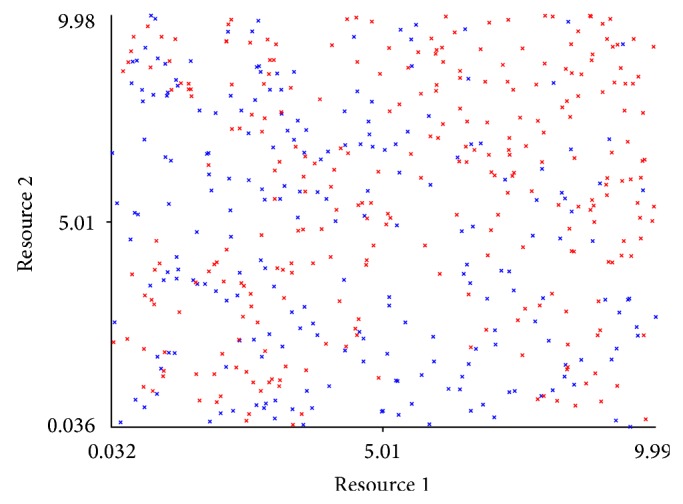
Initial distribution of two resources for stable and unstable instances.

We can see in Figure 9 the initial distribution of two resources for the case study with 3 agents. The resource of the third agent was omitted for simplicity. Each point in the figure is an instance of the system where the coordinates represent the initial values of the agent resources. The red instances represent the systems that became stable during execution, and the blue ones represent unstable ones. The visual aspect of the stable and unstable instances is entirely random. This figure provides a helpful intuition that it may not be possible to predict the stability or instability of a system a priori, only by looking at its initial conditions.

**Figure 10:**
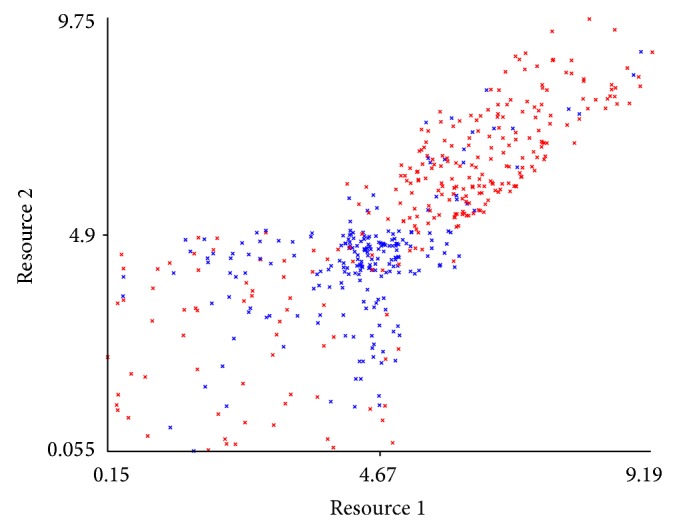
Final distribution of two resources for stable and unstable instances.

It is also interesting to inspect the distribution of the two resources after the multiagent system has been executed for a number of time steps (e.g., 10000), as shown in Figure 10. One can see that the values of the resources tend to be balanced in cases where their value is higher than the exchange threshold, that is, 5. In this seemingly diagonal region, many systems are stable, because the agents have enough exchange choices in order to avoid the situation when an agent *a* receives a resource unit from agent *b* but is unable to give anything back to *b*. This would cause a drop in the profit of *b* and also in its social model of *a*, leading in time to a different choice of interaction and thus causing the instability of the system. Much more unstable instances are therefore placed very close to the exchange threshold. This could be a clear indicator of instability, but unfortunately this is a postfactum consequence of the simulation and cannot be predicted before its start.

### 5.2. Transition Points

**Figure 11:**
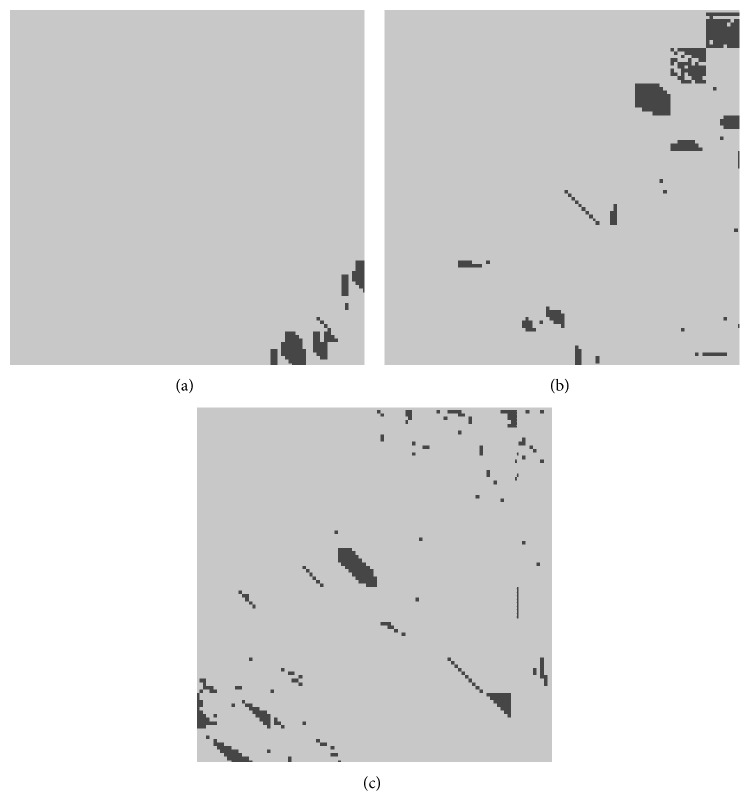
Continuous representation of stable and unstable systems in terms of resources *R*_1_ and *R*_2_, where (a) *R*_3_ = 0; (b) *R*_3_ = 5; and (c) *R*_3_ = 10.

Another line of investigation refers to the nature of phase transitions. Considering again a simple system with 3 agents and 1 resource, Figure 11 shows the stability (light grey) or instability (dark grey) of the system, based on the values of the resource. Unlike Figure 9, which only displayed sample points, Figure 11 displays a large number of initial values between 0 and 10, in order to identify regions of stability or instability. Since there are 3 values involved, we chose to represent 3 separate graphs, in which the abscissa corresponds to the resource of Agent 1 and the ordinate to the resource of Agent 2, and each graph corresponds to one of the following values of the resource of Agent 3: 0, 5, and 10, respectively. The graphs are not entirely symmetric because the needs of the agents are different.

Then, we tried to zoom in as closely as possible to the border between a stable region and an unstable region. Using the dichotomy technique, we could identify the values of a single resource for which the system would be stable (*v*_*s*_) or unstable (*v*_*u*_), with |*v*_*s*_ − *v*_*u*_| < 10^−6^. It was observed that the multiagent system does not exhibit gradual phase transitions. The behaviour of the system is quite similar when the resource value is either *v*_*s*_ or a stable value farther away from the border, compared to a resource value of either *v*_*u*_ or an unstable value farther away from it. Only the time steps when the swaps occur in case of instability can be somewhat different.

**Figure 12:**
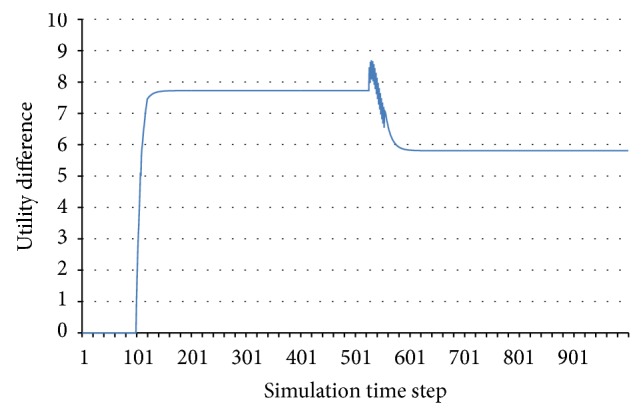
Difference between the sums of utilities with 10^−6^ difference in the resource of one agent.

We can observe this change in the situation with 3 agents and 1 resource. We can use the sum of utilities as a reference, to have a simpler image of the system behaviour.  Figure 12 shows the difference between two cases given by a change of only 10^−6^ in the resource of one agent, lying on both sides of the stability border and very close to it. One can notice that on the initial part of the simulation the difference is 0, but at some point the difference in resources causes an agent to make an alternative decision, and from then on the overall behaviour of the two systems becomes very different.

**Figure 13:**
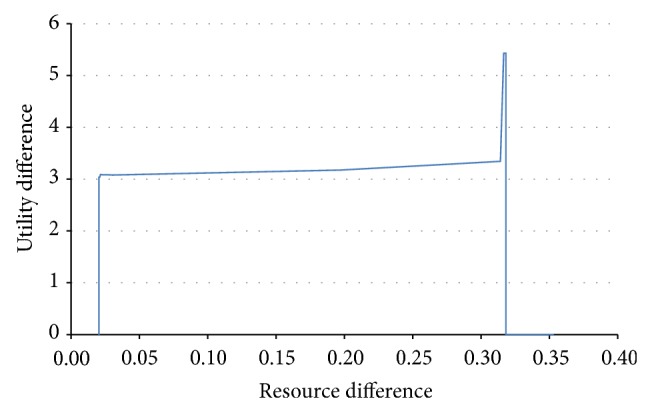
Maximum difference in a stable behaviour while traversing stable and unstable regions.

Let us consider now the same system configuration and see what happens when we make small changes in the resource of one agent, so as to cause the system to traverse a succession of stable–unstable–stable behaviours. In Figure 13, the ordinate represents the maximum difference between the sums of utility functions in the initial stable behaviour and in the situation where a resource is changed by the amount represented on the abscissa. More clearly, by changing the initial resource amount of one agent, we can cause the system to be stable or unstable. This is similar to traversing one of the subfigures in Figure 11 horizontally. At first, the system proves to be stable. In a subsequent run, when the resource amount is increased, it may enter an unstable area. By further increasing the resource, the system instance moves farther to the right and may enter a stable area again. We know how the sum of utilities varies in the initial, unchanged configuration. For each run, we also know the sum of utility functions. For each time step of the simulation, we can assess the difference between the sum of utilities in the unchanged system and the sum of utilities in the changed system. Figure 13 represents the maximum of those values, considering the entire simulation length. We can notice the sudden changes that appear when crossing the stability border, that is, when the difference in resource is around 0.02 and around 0.32.

By studying many similar situations, it was observed that the phase transitions of the multiagent system are abrupt, and changes between stable and unstable behaviours can be caused by minute changes in the internal states of the agents.

## 6. Stabilization Methods

Since our empirical analysis shows that it is impossible to find out whether a system with a given configuration will be stable or not, so as to be able to select only the stable ones, we propose three heuristic methods for the stabilization of a multiagent system during its execution.

### 6.1. Hybrid Model Stabilization

The first stabilization method, presented in Pseudocode 3, takes into account the “strong” criterion for stability, based on both internal models and external behaviour. By using the HybridStabilityTest procedure, it is assumed that we have identified an unstable behaviour and we apply the method only in this case. It is applied during the simulation after the transitory period and consists in small changes being made to the internal models of the agents.

The basic idea of the method is that if the difference of the social model outcomes decreases, it will eventually become 0 and will cause a swap. Therefore we can anticipate it and change the internal social model of the agent, in order to make it choose its second best peer sb for interaction. This alternative decision can be the right one and in this case the social model of sb will start to increase and the chance of stabilization will increase as well. Or it can be a poor decision and sb will provide less profit for the agent. In this case, given the small resulting difference between the social model outcomes of the two peers, eps (which can be, e.g., 10^−6^), if the second best is not good enough, the mistaken alternative decision will be quickly corrected.

Here and also in the next method, minDifference is one of the parameters which controls the moment when the actual perturbation of alternative decision making takes place. A value of 0 leads to very rare changes. The greater the minDifference, the higher the frequency of perturbations. However, a balance should be reached between the probability of stabilization and the simultaneous goal of achieving it in a minimally invasive way. It was observed in general that a higher value leads to a slight increase in the stabilization probability. For the following case studies, a value of 10^−3^ was used. Another parameter employed to control the number of changes is minStepsToChange. We used a value of 100 in the subsequent case studies, meaning that an agent model is swapped only if there have not been any other swaps affecting that agent in the previous 100 time steps.

### 6.2. Preemptive External Stabilization

The second stabilization method relies on the observation that many multiagent systems following the proposed protocol do not reach an internal stabilization but can nevertheless behave in a stable way for the duration of the simulation (and, in any case, for very long periods of time, if the difference between the social models outcomes decreases very slowly). The method is presented in Pseudocode 4.

The method implements an answer to the classical question “what if.” Since our multiagent systems are completely deterministic, we can simulate their evolution into the future, starting from any current state of the agents. Its basic idea is to verify that the system remains externally stable for some desired period of time into the future. Unlike the internal models, whose difference could provide some indications about possible swaps, the external behaviour must be observed for a prolonged period of time, to see if the system remains stable, because, as already stated, sudden changes could always appear after long periods of seeming stability. Observing the past behaviour is not enough to assess the future behaviour.

The method, which is more computationally intensive than the previous one, first makes a copy of the current state of the environment (including all the agents) and continues the simulation in this virtual environment for a number of desired time steps (e.g., 1000 or 10000). The endogenous perturbation mechanism is the same as in the first method. In every step of the simulation, or every 10 steps, to make it faster, the effects of the perturbation are verified. If the utility functions of the agents do not become constant or periodic in the virtual simulation, another change is made and so on.

The main* for* block in Pseudocode 4 is the same as in Pseudocode 3. The difference is that the test for stability is performed in the virtual environment, and the changes are applied only when instability is detected there.

### 6.3. Random External Stabilization

If the methods presented above fail, we can apply a more invasive way of stabilization. This method, presented in Pseudocode 5, is similar in a way to the mutation in evolutionary algorithms, which can get a solution out of a local optimum. Its basic idea is to reset the social models of the agents with a small probability in order to allow the creation of a new structure of interactions.

The same technique of checking forward the stability of the solution is applied. However, the perturbation mechanism is much more simple, because it does not take into account the social models of the agents. The social model outcomes of the agents are randomly reinitialized, and this gives them a chance to interact with different peers which may in turn lead to stabilization.

The test for external stability is the same as in Pseudocode 4; that is, it is performed in a virtual environment. However, the perturbation procedure is completely different, because it does not rely on the internal models, but it is randomly applied with a small probability.

### 6.4. Results and Discussion

In this section we assess the performance of the three proposed methods. Given their different nature, they are applied sequentially, trying the next only when the one before it, implementing a stronger stability criterion, fails. First, we attempt to stabilize the system using the hybrid criterion, which guarantees absolute stability. If it is not possible, we try a principled way to stabilize the external behaviour. If this also fails, we apply random perturbations in order to cause a different set of interactions which may bring about stability.

For the results presented below, 100 unstable multiagent systems were considered for each scenario, with different numbers of agents (3, 6, and 10) and resources (1, 2, 5, and 10). The success probability of a method was recorded. It must also be said that a small percentage of systems (less than 1%) could not be stabilized by running the three methods sequentially. However, they could be stabilized quite quickly by a second run of the third method.

In each scenario, the average number of steps after which the stabilization occurs (out of 10000 total time steps) and the average number of changes (as indicated by the noChanges variable in the pseudocode of the methods) were computed. The number of changes indicates the number of consecutive interventions during a single run before the system stabilizes. In most of the considered cases, the stabilized systems had constant values for the utility functions (period 1), and few exhibited a period of 2.

Tables 3, 4, and 5 display these results for each of the three methods.

If we consider the first scenario, with 3 agents and 1 resource, we can see that the first method is very effective. In fact, it is the only situation in which most systems can be internally stabilized. The probability of success of the first method decreases as the number of resources decreases. It can be noticed that the systems with 3 agents prove rather difficult to stabilize, once they settled into a structure of interactions. Actually, the agents have a very limited number of choices, since they can only decide between 2 peers. Consequently, only method 3 succeeds in stabilizing them for a larger number of resources. As the complexity of the systems increases, the second method becomes increasingly successful. These systems are difficult to stabilize internally, but they display more flexibility in terms of the number of choices of interaction an agent has. Most systems with 10 agents could be stabilized using the preemptive external method, while the random method was virtually no longer necessary.

One can also see that method 3 is less efficient in terms of stabilization speed. The average number of steps after which the systems become stable is the greatest out of the three methods, and also the number of changes to the system is the greatest. Since it relies on random perturbations rather than a definite principle of stabilization, different changes are tried until the system eventually stabilizes. This is why it is used as the last resort for stabilization, when the other two methods fail.

**Figure 14:**
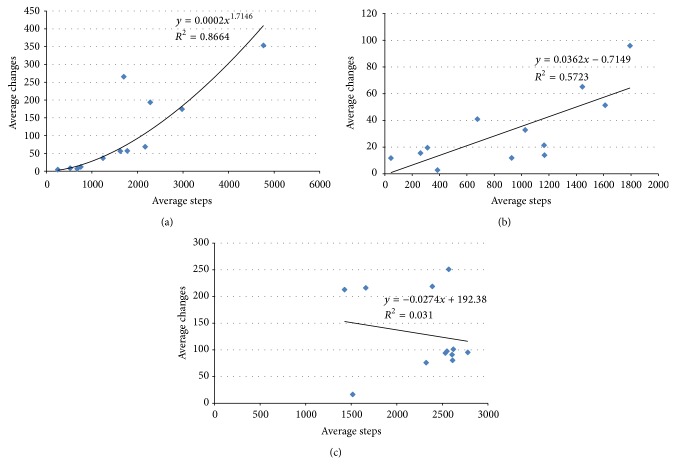
The variation of the number of changes with the number of steps until stabilization: (a) hybrid model; (b) preemptive external; and (c) random external.

Comparing the first two methods, we can see that they are similar in terms of average steps needed for stabilization. The second method seems more efficient though in terms of the number of changes to the system. It is also interesting to view the average number of changes as a function of the average number of steps needed for stabilization (Figure 14). For methods 1 and 2, there seems to be a relation between the two output measures; however, a relation does not seem to exist for the third, random method. The best fit corresponds to the first method, but the number of changes does not scale well when the number of steps needed for stabilization increases. The second method seems to be more efficient from this point of view, as well, even if there are quite large differences between the curve fitting line and the actual data.

## 7. Conclusions

The main contributions of this paper are the identification of the internal cause of instability in the multiagent systems following the proposed interaction protocol and the design of three methods that can be used to stabilize unstable systems. However, they are heuristic, and they can be applied successively, trying to satisfy stronger criteria first and ending with random perturbations, which are less efficient and more invasive but useful in case of less flexible configurations. Future work should investigate whether an algorithmic solution exists for this problem.

Another important issue is to identify an analytic model of phase transitions. Phase transitions are often studied in physical systems [70]; however, they have been demonstrated to occur in other agent-based systems, as well [71]. So far, there is only empirical evidence about the abrupt nature of phase transition in the multiagent systems considered in our paper. A formal proof would be needed as the next step. Also, it would be interesting to study whether one can identify a complexity index such as the rate of population growth in the logistic map and to see, based on this index, if patterns emerge when complexity increases, such as Feigenbaum constant which appears in the logistic map and some fractals, or other complexity patterns in cellular automata. In our case, such an index may likely depend on the number of agents, which adds an additional layer of difficulty to the problem.

Our research can be applied in agent-oriented computing and simulation. In a multiagent memetic system, similar to that presented in [72], which involves the exchange of resources among agents, the amount of resources gathered by the agents can become a basis for the evaluation of the agents. This can lead to the decision of reproduction or termination, allowing the inheritance of the genotype of agents in the system. Another application can be the modelling or enhancing of agent-based simulation of cooperation in a population of agents interacting in a spatial domain, which exchange resources and compete among themselves, for example, by playing the iterated prisoners' dilemma game [73].

Other applications could be found in social or economic simulations or for the modelling of biological processes. The concepts of stability and equilibrium are crucial aspects in all these domains, and therefore the idea of controlling such systems with small perturbations can have a great potential. The heuristics presented in this work are designed for deterministic complex systems; otherwise the stability test in a virtual environment which simulates future behaviour would be inapplicable. Another interesting investigation would be to study whether they can still be used for a more general class of multiagent systems, where determinism is not guaranteed, but shortterm predictions can still be made in an approximate form.

## Figures and Tables

**Pseudocode 1:**
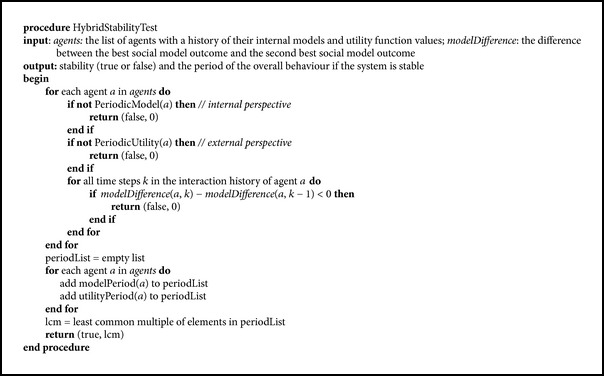
Hybrid test for stability.

**Pseudocode 2:**
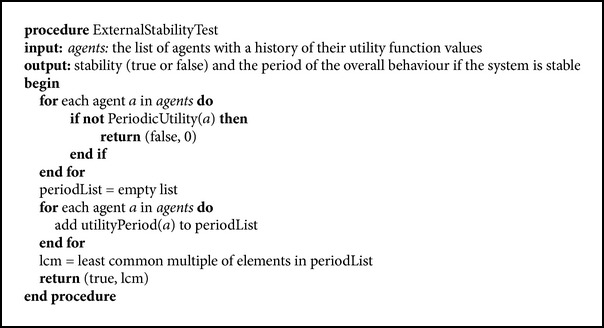
External test for stability.

**Pseudocode 3:**
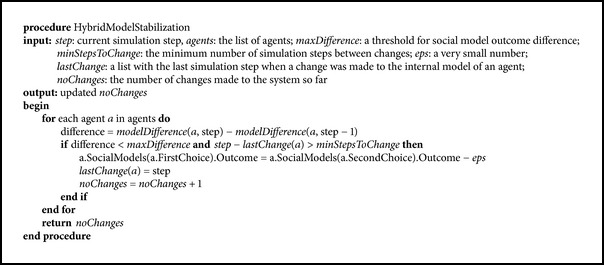
Hybrid model stabilization.

**Pseudocode 4:**
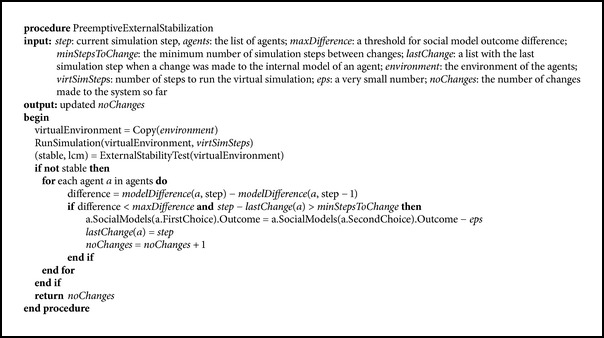
Preemptive external stabilization.

**Pseudocode 5:**
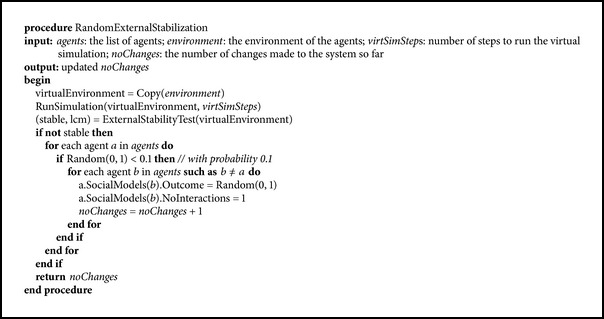
Random external stabilization.

**Table 1:** Classification results for the stability of a multiagent system with 3 agents and 1 resource.

Algorithm	Training	Cross-validation
C4.5	63.2%	60.2%
Random Forest	98.8%	80.6%
*k*NN	100%	76.8%
SVM, RBF	95.6%	83%

**Table 2:** Classification results for the stability of a multiagent system with 10 agents and 10 resources.

Algorithm	Training	Cross-validation
C4.5	89.3%	64.6%
Random Forest	99.2%	68.4%
*k*NN	100%	74.2%
SVM, RBF	100%	74.2%
SVM, Poly2	100%	66.8%

**Table 3:** Performance of the hybrid model stabilization method.

Agents	Resources	Success probability	Avg. steps	Avg. changes
3	1	82	248.95	4.42
3	2	30	522.98	8.29
3	5	14	754.93	12.11
3	10	9	677.56	6.72

6	1	27	1623.74	56.27
6	2	37	2168.37	68.65
6	5	41	1775.72	57.27
6	10	8	1242.56	36.64

10	1	14	2279.35	193.60
10	2	13	2977.03	174.57
10	5	11	4765.13	353.33
10	10	11	1700.17	265.45

**Table 4:** Performance of the preemptive external stabilization method.

Agents	Resources	Success probability	Avg. steps	Avg. changes
3	1	13	385.37	2.83
3	2	38	927.39	11.92
3	5	36	1167.44	13.94
3	10	12	1164.23	21.42

6	1	68	44.14	11.78
6	2	61	311.97	19.55
6	5	58	1026.18	32.88
6	10	75	1610.95	51.36

10	1	85	261.04	15.55
10	2	86	676.85	40.97
10	5	88	1444.33	65.12
10	10	88	1792.74	95.90

**Table 5:** Performance of the random external stabilization method.

Agents	Resources	Success probability	Avg. steps	Avg. changes
3	1	5	1515.72	16.58
3	2	32	2322.16	76.12
3	5	50	2611.80	80.47
3	10	79	2606.61	91.09

6	1	5	2532.47	94.14
6	2	2	2779.04	95.38
6	5	1	2550.06	97.64
6	10	17	2619.98	101.32

10	1	1	1426.30	212.93
10	2	1	1659.65	216.18
10	5	1	2389.85	218.87
10	10	1	2569.67	250.91
